# Factors Affecting Date of Implantation, Parturition, and Den Entry Estimated from Activity and Body Temperature in Free-Ranging Brown Bears

**DOI:** 10.1371/journal.pone.0101410

**Published:** 2014-07-02

**Authors:** Andrea Friebe, Alina L. Evans, Jon M. Arnemo, Stéphane Blanc, Sven Brunberg, Günther Fleissner, Jon E. Swenson, Andreas Zedrosser

**Affiliations:** 1 Department of Biosciences, Biologicum, Institute of Cell Biology and Neurosciences, Goethe University Frankfurt, Frankfurt/M., Germany; 2 Department of Forestry and Wildlife Management, Hedmark University College, Campus Evenstad, Elverum, Norway; 3 Department of Forestry and Wildlife Management, Faculty of Applied Ecology, Agricultural Sciences, Hedmark College, Campus Evenstad, Elverum, Norway; 4 Département Ecologie, Physiologie et Ethologie, Institut Pluridisciplinaire Hubert Curien, UMR 7178 CNRS/UDS, Strasbourg, France; 5 Department of Ecology and Natural Resources Management, Norwegian University of Life Sciences, Ås, Norway; 6 Norwegian Institute of Nature Research, Trondheim, Norway; 7 Department of Environmental and Health Studies, Faculty of Arts and Science, Telemark University College, Bø i Telemark, Norway; 8 Institute for Wildlife Biology and Game Management, University for Natural Resources and Life Sciences, Vienna, Austria; 9 Department of Wildlife, Fish and Environmental Studies, Faculty of Forest Sciences, Swedish University of Agricultural Sciences, Umeå, Sweden; University of Missouri, United States of America

## Abstract

Knowledge of factors influencing the timing of reproduction is important for animal conservation and management. Brown bears (*Ursus arctos*) are able to vary the birth date of their cubs in response to their fat stores, but little information is available about the timing of implantation and parturition in free-ranging brown bears. Body temperature and activity of pregnant brown bears is higher during the gestation period than during the rest of hibernation and drops at parturition. We compared mean daily body temperature and activity levels of pregnant and nonpregnant females during preimplantation, gestation, and lactation. Additionally we tested whether age, litter size, primiparity, environmental conditions, and the start of hibernation influence the timing of parturition. The mean date of implantation was 1 December (SD = 12), the mean date of parturition was 26 January (SD = 12), and the mean duration of the gestation period was 56 days (SD = 2). The body temperature of pregnant females was higher during the gestation and lactation periods than that of nonpregnant bears. The body temperature of pregnant females decreased during the gestation period. Activity recordings were also used to determine the date of parturition. The parturition dates calculated with activity and body temperature data did not differ significantly and were the same in 50% of the females. Older females started hibernation earlier. The start of hibernation was earlier during years with favorable environmental conditions. Dates of parturition were later during years with good environmental conditions which was unexpected. We suggest that free-ranging pregnant brown bears in areas with high levels of human activities at the beginning of the denning period, as in our study area, might prioritize investing energy in early denning than in early parturition during years with favorable environmental conditions, as a strategy to prevent disturbances caused by human.

## Introduction

Embryonic diapause, a widespread strategy to ensure and optimize successful reproduction, is common in plants, insects, fish, birds, and mammals [1], [2]. Diapause and delayed implantation involve several independently controlled steps and many of the biological processes are still poorly understood [3]. Bears are the only mammals with delayed implantation, gestation, parturition, and lactation during hibernation, when they do not eat, drink, urinate, or defecate for several months. During this period they survive solely on their stored energy resources [4]–[6].

Gestation in ursids lasts approximately 60 days [7]–[9]. This short period limits the energetic costs of reproduction by truncating embryonic development, which in turn reduces the size of offspring and thus the initial costs of lactation [8], [10]. The gestation period of bears has been estimated mainly with macroscopic and histological investigations of the ovaries and uteri of hunter-killed females or with blood serum analysis in captive and free-ranging bears [7], [8], [11]. Quest [9] determined a 54-56-day gestation period in captive brown bears using ultrasonic examination.

Examinations of the reproductive organs of free-ranging and captive brown (*Ursus arctos*) and American black bears (*U. americanus*) indicate that implantation occurs in late November to early December, and parturition occurs in late January to early February [11]–[16]. Studies of serum plasma progesterone concentrations of pregnant and nonpregnant female American and Asiatic black bears (*Ursus thibetanus*) and brown bears gave similar results [17]–[20]. The time of parturition has also been determined for American black bears by listening for vocalizations of cubs at the den sites [21], [22].

Many aspects of the reproductive biology of ursids are still poorly understood, such as reproductive cycles, hormone and estrous cycling, and factors that trigger implantation and birth. Most of these studies have been carried out in captivity [8], [23], [24] and little information is available about the timing of implantation and parturition in free-ranging bears. The reproduction biology of ursids is controlled by a complex timing system, in which the chronological sequence is determined by seasonality [8], [25]. Although photoperiod is an important regulator of the reproductive cycle, the mating season and the duration of embryonic diapauses vary among ursid species and individuals [8], [25], [26]. The mating season of most bear species occurs in spring or early summer and lasts approximately 2–2.5 months. Fertilized eggs undergo diapause at the blastocyst stage for 4–5 months until delayed implantation occurs [11], [17], [19], [20]. The duration of embryonic diapauses varies, because the time of implantation and birth is uncoupled from the mating season [8], [10]. Cubs in a litter are normally born at the same date independently of the dates of estrus and mating [8], [10], [12]. Split parturition has been observed in a captive brown bear, but has not been documented in the wild [27].

Several studies of bears have shown a strong correlation between a females' body condition in fall and their reproductive success. Well-nourished females have larger litter sizes and shorter litter intervals [28]–[33]. A minimum amount of body mass and fat content (19% in brown bears) prior to hibernation is necessary for reproduction [34]–[38]. Thus, brown bears are able to vary both the birth date and growth rate of their cubs in response to their fat stores, which means that females in superior condition give birth earlier and lactate longer and produce more and higher quality milk in the den than females in poorer condition. This also accelerates cub growth relative to females in poorer condition [37], [39]. Knowledge about the timing of reproductive events is therefore important for conservation and management.

Our first aim was to document, for the first time, the dates of implantation, parturition, and the gestation period of free-ranging brown bears. Embryo development requires euthermia and the body temperature of pregnant female brown and black bears is higher (∼37°C) during the gestation period than during the rest of hibernation (32–34°C). Body temperature drops at parturition [40]–[43]. We used the rise and drop in body temperature of pregnant females to calculate the dates of implantation and parturition and to document the gestation period of free ranging brown bears in Sweden. We compared the body temperature of pregnant females before, during, and after the gestation period and also with the body temperature of nonpregnant females.

Hibernating pregnant female brown bears are more active during pregnancy than afterwards. Their activity levels increase at the end of November, remain elevated, and then drop sharply to a lower level in late January/early February, similar to the progress of body temperature that is reported for pregnant females during hibernation [44]. We compared if activity data (recorded in GPS collars) and body temperature data (recorded in implanted temperature loggers) would yield the same dates of implantation or parturition.

Our second aim was to determine which factors influence the timing of gestation. We tested whether age, litter size, primiparity, environmental conditions during season before hibernation, or the date of the start of hibernation influence the timing of parturition. In addition, we evaluated whether age, primiparity, environmental conditions, or weather conditions in autumn influence the start of hibernation.

## Methods

### Study area

The study area was located in the northern boreal forest zone in Dalarna and Gävleborg counties, south-central Sweden (∼61°N, 15°E). The area is hilly, with altitudes ranging from 200 m in the southeast to 1,000 m in the west, but are mostly (>90%) below timberline, which is at ∼750 m [45]. Snow cover usually lasts from the end of October until late April, and mean daily temperatures range from −7°C in January to 15°C in July (Swedish Meteorological and Hydrological Institute). The bear population density is ∼30/1000 km^2^ [46], [47]. The denning period in in the study area is from October until May, and its duration varies due to reproductive status. Pregnant females spend on average 196 days in den, about one month longer than nonpregnant bears in the study area [48], [49]. Timing of den entry is influenced by sex, reproductive status, and environmental conditions (e.g. first snowfall), as well as age and/or body size [48], [49]. Pregnant females enter their dens first and leave their dens latest [48].

### Capture, sensors, and the bears

We captured bears in spring after they left their dens. For detailed capture and marking procedures, see Arnemo et al. [50]. The permission to capture and instrument bears was granted by the Swedish Environmental Protection Agency (permit Dnr 412-7327-09 Nv) and the Ethical Committee on Animal Experiments in Uppsala (approval C47/9). Every bear was equipped with a dual-axis motion sensor mounted on a GPS-GSM collar (Vectronic Aerospace GmbH, Berlin). This sensor measures true acceleration six to eight times per second in two orthogonal directions. The acceleration values were accumulated and averaged for each direction for a recording interval of 5 minutes, resulting in average acceleration values ranging from 0 to 255 for each axis. These averaged acceleration values were stored in the neck collar with the associated date and time until they were downloaded as a text file via Link Manager (Vectronic Aerospace GmbH, Berlin). We implanted abdominal temperature data loggers (DST Centi, Star Oddi, Iceland), programed to record body temperature every 30 minutes (see Arnemo et al. [50] for further details on the implantation procedures). These temperature data were stored in the logger's internal memory with a real-time clock reference for each measurement. After recapturing the bears, we recovered the temperature loggers and uploaded the body temperature data with SeaStar software and the Communication Box (Star Oddi, Iceland), which served as a wireless interface between the logger and a PC.

Only females with verified reproductive status in a given year were included in the data set. Pregnant females were defined as solitary-hibernating females that had been observed with cubs of the year (hereafter referred to as cubs) after den emergence in spring, or which had been captured shortly after den emergence and showed signs of lactation and that cubs had used the nipples (to exclude cases of pseudopregnancy). Females were defined as nonpregnant when they had emerged from the den without cubs and showed no signs of lactation when captured. We defined the hibernation period as 1 November–31 March and calculated the mean daily body temperature and mean daily activity during this hibernation period for all females, based on the methods described by Friebe et al. [44]. It is common that bears abandoned their first dens (∼22% of the cases), mainly as a result of human disturbance [51]–[53]. Two of our bears changed dens at the end of October and entered new dens in early November. For those bears we chose the second den entry as the start of hibernation.

### Definition of the gestation period

#### Body temperature data

The body temperature T(b) of pregnant females bears is on average higher and more stable during the period of gestation than that of nonpregnant females [40], [41]. After parturition, T(b) drops to the level of nonpregnant bears [41], [42]. We defined the hibernation period as 1 November until 31 March [48], [49] and calculated the mean body temperature during hibernation for each individual. The date of implantation was defined as the first day in November/December when an individual's mean daily body temperature exceeded the same individual's mean temperature during hibernation. Occasional high body temperature recordings, apparently caused by external factors, e.g. disturbances during hibernation, were excluded from the data set [40], [41], [54]. We defined the date of parturition as the first day in January/February when an individual's mean daily body temperature declined below the individual's mean temperature during hibernation. The gestation period was defined as the time interval between the dates of implantation and parturition.

#### Activity data

Bears are inactive ∼98% of the time during hibernation, but they periodically make small movements [41], [44], [55], [56]. Therefore, only a few position movements may have a large impact on the mean daily activity level. Robbins et al. [57] observed that pregnant captive brown bears did not stand up during the first 3 weeks postpartum. However, Friebe et al. [44] observed that some females have this low activity level for a shorter time after parturition. We therefore defined the date of parturition as the first day when the individual's mean activity level decreased below the same individual's mean hibernation activity level for at least 2 weeks. In central Sweden, 22% of the brown bears change winter dens, most often early in the denning period, when human hunting activities are still high [53]. Activity levels during hibernation are also lowest during midwinter [44], [55]. For these reasons, occasional high activity peaks often occur early in hibernation, when implantation also occurs. To minimize the effect of high activity peaks, we used the moving averages (5^th^ order) of the mean daily activity levels when defining the dates of implantation. The first day when this moving average exceeded the individual's mean hibernation activity level for at least the next 2 weeks was defined as the day of implantation.

We used the dates of implantation and parturition calculated with body temperature data to compare the recorded activity and body temperature data during the gestation period with that obtained from two other periods: 14 days before gestation (preimplantation period) and 14 days after parturition (lactation period). We used relatively short periods of 14 days, because we wanted to compare data collected only during the hibernation period. Implantation may occur some weeks after the start of hibernation, and the time in den during lactation may be short for females that give birth to cubs very late. The body temperature of hibernating nonpregnant American and Asian black bears show multiday cycles, whereas pregnant females remain normothermic during gestation [41], [42]. We compared the mean body temperature and also the daily variation in body temperature during the preimplantation, gestation, and lactation periods for pregnant and nonpregnant bears. For nonpregnant bears, we used the mean date of implantation and parturition determined from pregnant bears with body temperature recordings to define the periods of preimplantation, gestation, and lactation. Activity levels of pregnant and nonpregnant bears has been compared in a previous study [44].

### Factors influencing date of birth and start of hibernation

Maternal body condition prior to denning influences reproductive success in bears [39]. Because we did not capture bears in autumn or winter, we had no information about the maternal body mass or fat content in autumn, nor information about cub growth. Instead, we calculated a yearling condition index for each year, which reflects the combined effect of environmental factors on the bear's condition. The environmental condition index had been used in former studies as a proxy for food conditions [58]. We regressed the spring yearling body mass of 307 yearlings as a function of maternal size, litter size, population density, and sex, variables that are known to influence yearling mass independently of environmental conditions. The standardized residual values from this regression were averaged for each year and used as the environmental condition index for the previous year, when the yearlings had been cubs [58]. We then tested whether the environmental condition index, age, primiparity, litter size, and the start of hibernation influenced the date of parturition.

Harsh climate and weather conditions may trigger the start of hibernation and prolong the duration of denning [59]–[61]. Additionally it has been reported that black and brown bears in excellent condition start hibernation earlier [28], [61], [62]. We created individual activity indices by summing the acceleration values on the orthogonal axes (0–510) for each 5-minute interval. A bear was considered to be physically active when its activity index was higher than 22.9 [63]. The start of hibernation was defined as the first day in autumn when activity dropped below 1 hour per day (defined as fewer than 12 activity recordings with levels > 22.9 per day) [43], [53]. The mean ambient temperature in October was used as the index for the weather conditions during the period of hibernation start.

### Data analysis

We tested for relationships between mean daily activity and mean daily body temperature during hibernation with a general linear mixed model with normal distribution and with individual identity as a random factor. A second-order polynomial term for mean daily body temperature was included into this analysis to account for nonlinear effects. We used paired-samples t-tests to compare the dates of implantation and parturition and the gestation period between the estimates based on activity and body temperature data. Activity and body temperature data during the preimplantation, gestation, and lactation periods were compared with paired Wilcoxon signed rank tests. To evaluate the effect of the day of gestation on body temperature, we used a general linear mixed model with a normal distribution and with individual identity as a random factor. A second-order polynomial term for day of gestation was included into this analysis to account for nonlinear effects. Mann Whitney U tests (MWU) were used to compare body temperatures during preimplantation, gestation, and lactation periods between pregnant and nonpregnant females. We evaluated the factors affecting the date of parturition with a general linear mixed model with a normal distribution and assessed the effects of the following factors: age, primiparity (as binomial variable, with no = 0; yes = 1), litter size, date of hibernation start, and the environmental condition index. Because some mothers contributed several litters to our datasets during their lifetime, we included individual identity as a random effect to account for nonindependence. Year was not included as a random effect, because the environmental condition index was included as a fixed variable to describe the different environmental conditions among years. We used a backward procedure to select the best models, based on P values with a significance level of α = 0.05, starting with a full model of all covariates and relevant second-order interactions.

We used a linear mixed model with a normal distribution to evaluate the effects of age, primiparity, and environmental condition indices on the start of hibernation, with individual identity as random effect. Ambient temperature in October was excluded from the model, because of collinearity with the environmental condition index (Pearson's r: −0.745, P<0.001). We used a linear mixed model with a normal distribution to evaluate the effects of mean ambient temperature in October on the start of hibernation, with individual identity as a random effect. A linear mixed model with a normal distribution was also used to evaluate the effects of age and environmental condition index on the duration of hibernation prior to parturition. Residuals from all final models were inspected visually to ensure that the assumptions of constancy of variance and normality of errors were met. All statistical tests were carried out in SPSS (PASW Statistics 21).

## Results

We compared body temperature data from 9 hibernation events from 8 nonpregnant adult females during 2010–2013, and body temperature and activity data from 6 hibernation events from 4 pregnant females, during 2010–2013.

### Body temperature

**Figure 1:**
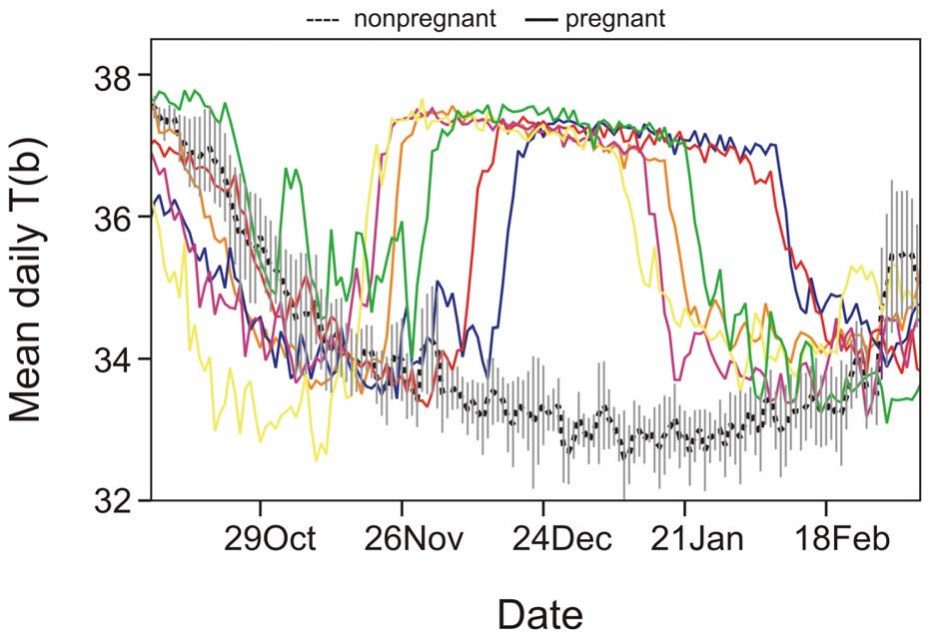
Mean daily body temperature (T(b)) of pregnant (N = 6) and nonpregnant (N = 9) hibernating female brown bears in Sweden, during 2010–2013. The solid lines show the mean daily T(b) of 6 individual pregnant females, the dotted line shows the mean daily T(b) of 9 nonpregnant females, including the daily SE (gray bars). The T(b) decreased throughout gestation (Estimate = −0.002, SE<0.001, P<0.001).

**Figure 2:**
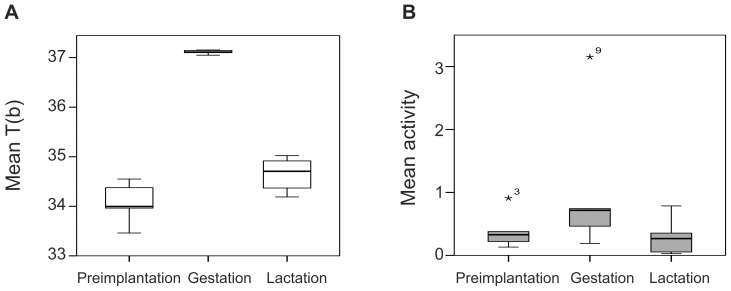
Mean body temperature (T(b)) (A) and mean activity (B) during preimplantation, gestation, and lactation periods for pregnant females brown bears (N = 6) in Sweden, during 2010–2013. We calculated the dates of implantation and parturition with body temperature data. Preimplantation was defined as the 14-day period before implantation occurred and lactation was defined as the 14-day period after parturition. Extreme outliers are plotted as asterisks. In figure B, the highest activity values in all periods originate from the same pregnant female.

**Figure 3:**
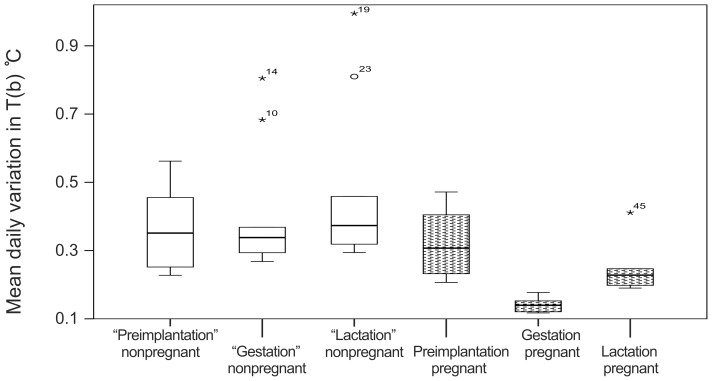
Daily variation in body temperature (T(b)) during preimplantation, gestation, and lactation for pregnant (N = 6) and nonpregnant (N = 9) female brown bears in Sweden during 2010–2013. For nonpregnant bears, we used the mean date of implantation and parturition determined from pregnant bears with body temperature recordings to define the periods of preimplantation, gestation, and lactation. The box indicates the median, 25, and 75% percentiles; the whiskers show the minimum and maximum observed values that are not statistically outliers. The extreme outlier is plotted as an asterisk.

The body temperature of pregnant females increased in November/December and remained high until January/February (Fig. 1). Based on body temperature, the estimated mean date of implantation was 1 December ±12 days (median: 28 November, range: 29 days, from 19 November – 18 December). The mean date of parturition was 26 January ±12 days (median: 23 January, range: 29 days, from 12 January – 10 February). The mean duration of the gestation period was 56 ±2 days (median: 56 days, range: 54 – 59 days) (Table 1). Mean body temperature during the preimplantation, gestation, and lactation periods was 34.07±0.42°C (median: 34.00°C), 37.11±0.04°C (median: 37.11°C), and 34.64±0.32°C (median: 34.71°C), respectively. We excluded the preimplantation period of one pregnant female that had shifted den 3 days before implantation from the analysis. Mean body temperature was significantly higher during the gestation period than during both the preimplantation and the lactation periods (paired sample Wilcoxon test: preimplantation vs gestation: Z = 2.02, P = 0.043; gestation vs lactation: Z = −2.20, P = 0.028) (Fig. 2A). We found no significant difference in body temperature between the preimplantation and lactation periods of pregnant females (Wilcoxon signed rank test: Z = −1.75, P = 0.080; Fig. 2A)). The body temperature of pregnant females decreased during the gestation period (Estimate = −0.002, SE<0.001, P<0.001; Fig. 1). The mean body temperature of nonpregnant bears during the time period corresponding to the preimplantation period of pregnant females was 33.85±0.54°C (median: 33.97°C), during the corresponding gestation period 33.19±0.34°C (median: 33.18°C), and during the corresponding lactation period 33.06±0.36°C (median: 33.05°C). The body temperature of pregnant females was significantly higher during the gestation and lactation periods than that of nonpregnant females during the corresponding periods (MWU; gestation: U = 54, P<0.001; lactation: U = 54, P<0.001). We found no significant difference in body temperature among pregnant and nonpregnant females during the preimplantation period (MWU; U = 29, P = 0.438). The mean daily variation in body temperature for pregnant and nonpregnant bears was 0.32±0.10°C (median: 0.31°C) and 0.36±0.12°C (median: 0.35°C), respectively, during the preimplantation period, 0.14±0.02°C (median: 0.14°C) and 0.41±0.19°C (median: 0.34°C), respectively, during the gestation period, and 0.25±0.08°C (median: 0.23°C) and 0.48±0.25°C (median: 0.37°C), respectively, during the lactation period. The mean daily variation in body temperature during the gestation period was lower for pregnant females than all other periods for pregnant and nonpregnant females (MWU: gestation(preg) vs preimplantation(preg) U = 0, P = 0.002; gestation(preg) vs lactation(preg) U = 36, P = 0.002; gestation(preg) vs all periods(nonpreg); U = 0, P>0.001). The mean daily variation in body temperature during the lactation period for pregnant females also was lower than all periods for nonpregnant females (MWU: lactation(preg) vs preimplantation(nonpreg) U = 10, P = 0.050; lactation(preg) vs gestation(nonpreg) U = 7, P = 0.018; lactation(preg) vs lactation(nonpreg) U = 6, P = 0.012). There were no significant differences between the mean daily variation in body temperature during preimplantation among pregnant and nonpregnant females (MWU: U = 23, P = 0.689; Fig. 3).

**Table 1:** Gestation periods of 6 pregnant female brown bears in Sweden, during 2010–2013, calculated from body temperature (T(b)) and activity data.

		Date	Difference	Date	Difference	Gestation	Difference
Bear Id-Year-Age		Implantation	(days)	Parturition	(days)	(days)	(days)
W0820-12-6	T(b)	30 Nov		25 Jan		56	
	activity	01 Dec	1	24 Jan	1	54	−2
W0605-11-7	T(b)	19 Nov		12 Jan		54	
	activity	11 Nov	−8	12 Jan	0	62	8
W0703-11-6	T(b)	25 Nov		20 Jan		56	
	activity	12 Nov	−13	20 Jan	0	69	13
W0610-11-7	T(b)	18 Nov		10 Feb		54	
	activity	01 Dec	−17	09 Feb	−1	70	16
W0610-12-8	T(b)	11 Dec		8 Feb		59	
	activity	02 Dec	−9	8 Feb	0	68	9
W0720-11-12	T(b)	21 Nov		16 Jan		56	
	activity	22 Nov	1	18 Jan	2	57	1
Total mean	T(b)	01 Dec		26 Jan		55.8	
Total median	T(b)	28 Nov		23 Jan		56	
Total SD	T(b)	11.6		12.0		1.8	
Total Range	T(b)	29		29		5	
Total mean	activity	24 Nov		26 Jan		63.3	
Total median	activity	27 Nov		23 Jan		65	
Total SD	activity	9.7		11.5		6.7	
Total Range	activity	21		28		16	

### Activity data

**Figure 4:**
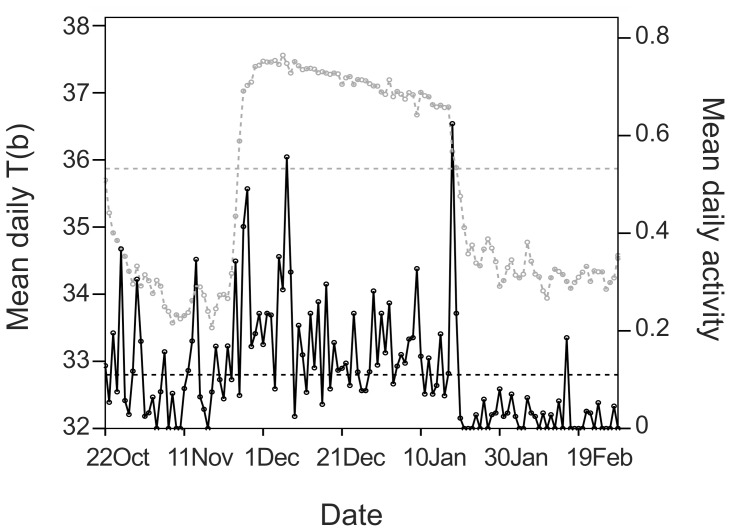
Example of the mean daily activity and mean daily body temperature (T(b)) recordings for a hibernating pregnant female brown bear in Sweden. The horizontal gray and the black dotted lines show the mean individual body temperature and activity during hibernation, respectively, which were used to calculate the dates of implantation and parturition.

The activity and body temperature data of 6 pregnant females showed similar patterns (Fig. 4). Mean daily activity and body temperature were positively related during the defined hibernation period (estimate = 0.205, SE = 0.021, t = 9.865, P<0.001). The implantation dates we estimated based on activity data differed up to 17 (±5) days from the implantation dates we estimated based on body temperature, but the means were only barely statistically equal (paired-sample t test: t = 2.512, df = 5, P = 0.054). The calculated parturition dates differed only by a maximum of 2 days, which was not statistically significant (paired-sample T-test: t = 0.000, df = 5, P = 1.000). The calculated gestation periods based on activity data were significantly longer than those based on body temperature data (paired-sample T-test: t = −2.667, df = 5, P = 0.045; Table 1). Mean activity during preimplantation, gestation, and lactation periods was 0.39±0.3 (median: 0.33), 1.0±1.08 (median: 0.72), and 0.29±0.27 (median: 0.27), respectively. Activity during the gestation period was significantly higher than during both the preimplantation and the lactation periods (paired-sample Wilcoxon test; preimplantation vs. gestation: Z = 2.02, P = 0.043; gestation vs. lactation: Z = −2.20, P = 0.028; Fig. 2B). Activity during the lactation period was significantly lower than during the preimplantation (paired-sample Wilcoxon test: Z = 2.02, P = 0.043; Fig. 2B).

We used a data set of 46 hibernation events from 30 females with only activity data to investigate which factors influence the date of parturition and the start of hibernation. The mean age of the females during these 46 hibernation events was 9.0±4.0 years (range: 16, from 3 – 19 years). Eleven females were primiparous, 33 were multiparous, and in 2 cases the previous reproductive status could not be classified. Mean litter size after hibernation was 2.16±0.74 cubs. Mean date of parturition was 21 January (SD: 9, median: 21 January, range: 43 days, from 1 January – 13 February). The mean start of hibernation was 18 October (SD: 9, median: 16 October, range: 34 days, from 2 October – 5 November). The mean duration of denning prior to parturition was 95 days (SD: 13, median 93, range: 57 days, from 66 – 123 days).

**Figure 5:**
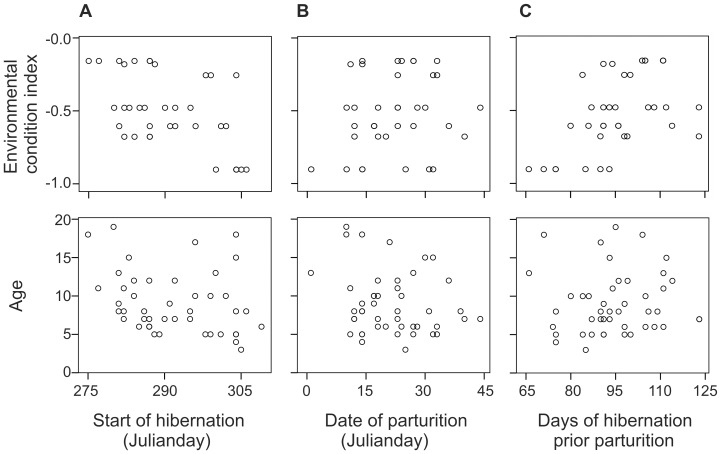
The effect of age and environmental conditions on the start of hibernation (A), date of parturition (B) and on the days of hibernation prior parturition (C) for 46 hibernating pregnant female brown bear in Sweden. The environmental condition index was significant related to the start of hibernation, date of parturition and on days of hibernation prior parturition. Age was significantly related only to the start of hibernation.

The start of the hibernation was earlier when ambient temperatures in October were low (estimate = 2.064, SE = 0.47, t = 4.394, P<0.001). Older females started hibernation earlier (estimate = −0.654, SE = 0.31, t = −2.09, P = 0.044) and the start of hibernation was earlier when environmental conditions had been positive (estimate = −12.243, SE = 3.10, t = −3.95, P<0.001) (Fig. 5A). Nonsignificant variables were excluded from the linear mixed model in following order: litter size (β = −0.121, t = −0.658, P = 0.516), primiparity/multiparity (β = −0.144, t = −0.945, P = 0.350).

The date of parturition was later when environmental conditions had been positive (estimate = 8.112, SE = 3.61, t = 2.250, P = 0.030). Nonsignificant variables were excluded from the linear mixed model in following order: start of hibernation (β = −0.010, t = −0.063, P = 0. 950), primiparity/multiparity (β = −0.008, t = −0.054, P = 0.957), age (β = −0.198, t = −1.338, P = 0.188), litter size (β = 0.032, t = 0.171, P = 0.866, Fig. 5B). The duration of hibernation prior to parturition was longer when environmental conditions had been positive (estimate = 20.204, SE = 4.82, t = 4.202, P<0.001). Age was not related to the length of duration of hibernation prior to parturition (β = 0.051, t = 0.334, P = 0.740, Fig. 5C)

## Discussion

### Body temperature

This is the first time the timing of gestation has been documented in free-ranging brown bears. The body temperature data clearly identified the dates of implantation and parturition. The calculated gestation periods ranged between 54 – 59 days and were similar to early reports for black and brown bears in other studies. Body temperature averaged higher during the gestation period compared to the preimplantation and lactation periods for pregnant females and compared to the body temperature of nonpregnant female bears. Besides the energetic costs of lactation, the maintenance of a high body temperature during gestation may be an additional reason why pregnant females loose more body mass during hibernation than nonpregnant bears [38]. The mean daily body temperature during gestation varied very little compared to the periods before and after the gestation and compared to the body temperature of nonpregnant females. Multiday cycles of body temperature have been documented for nonpregnant hibernating bears [41], [64]. We did not observe this in pregnant females during the gestation period. Instead, the mean daily body temperature was stable and did not fall below 35.9°C, as also observed in one pregnant American black bear [41]. Fetal development might be intolerant of high variations in body temperature. Raised hormone levels during pregnancy could be another reason for the low variation of body temperature during gestation [7].

We also observed that the body temperature of pregnant females decreased during the course of gestation. Studies have shown that the maximum serum progesterone level of pregnant brown bears occurs approximately 60 days before parturition and decreases during gestation [7]. The decrease in body temperature during gestation that we observed might be caused by changes in progesterone or other hormone levels.

A drop in body temperature at parturition has been reported previously for American and Asiatic black bears and brown bears; in both species of black bears, the body temperature decreased to the level of nonpregnant bears after parturition [41], [42]. However, our results for brown bears showed that the body temperature during lactation did not fall as low as that of nonlactating bears, as also reported by Hissa [40] for brown bears. Our data showed that body temperature during the preimplantation period did not differ significantly from that during lactation for pregnant females. However, nonpregnant females had lower body temperature levels than pregnant females during the lactation period. Body temperature is probably lowest during midwinter, as it is for activity [40], [44]. Metabolic activity during lactation might require or result in higher body temperature levels.

### Activity

Parturition dates estimated using activity and body temperature data differed by only one or two days and were the same for 50% of the females. Thus, we consider that either activity data or body temperature can be used to determine dates of parturition. However, because of the high variation in mean daily activity during the early hibernation period, it was more difficult to estimate the dates of implantation. We used the moving 5^th^-order average, because in some cases, activity did not reach the mean hibernation level for more than a few days before implantation, in other cases activity rose before the implantation calculated from the body temperature. Raised activity during this period could be caused by hormonal changes prior implantation, or because activity is in general higher during the beginning of the hibernation period than during midwinter [44], [55]. Our calculated dates of implantation varied 17±5 days between body temperature and activity recordings. We can therefore not recommend using activity recordings to determine the date of implantation. However, because the gestation period was stable, showed little variation and lasted on average 56 days, we recommend estimating the date of implantation using activity data by subtracting 56 days from the calculated date of parturition.

### Factors that influenced the date of parturition

Parturition dates ranged over a period of 43 days, which showed a high flexibility in the timing of gestation. Whereas dates of parturition have not been recorded in wild-living brown bears before, Bridges et al. [65] documented that parturition dates of 150 litters of wild-living American black bears ranged over 53 days from late December to mid-February (39 days excluding an outlier). Robbins et al. [39] reported only a 17-day range of parturition in January for a smaller sample of 13 captive brown bear births, perhaps due to similar conditions between bears in captivity.

Because the date of denning did not correlate with the date of parturition, we suggest that other factors than the start of denning trigger implantation. Age had no significant effect on the timing of parturition, as there was only a tendency for older females to give birth earlier. Bridges et al. [65] observed later parturition in pregnant female American black bears < 5 years old. However, in our study, only 2 females were < 5 years. A larger dataset of young pregnant females might be necessary to document an effect of age on the date of parturition.

Studies on captive brown bears have shown that larger females give birth earlier during winter than smaller females [39]. However, in our study, favorable environmental conditions correlated with late parturition. Although we had no information about the females' body mass prior to denning, we expected that food availability was the most important factor affecting the environmental condition index [58] and that the females were heavier when the environmental conditions had been favorable. With this reasoning, our results differed from those found in captive bears [39]. It is possible that free ranging females might budget their energy resources differently than captive females [66], [67].

### Timing of the start of hibernation

The start of hibernation varied 34 days, with a mean start of 18 October, similar to previous studies in our study area [48], [53]. Good environmental conditions were highly significantly correlated with an earlier start of hibernation. Early start of hibernation has been observed as a strategy for extremely well nourished female bears [61]. Limited fat-storing capacity can be a reason for early start of hibernation during years with good environmental conditions [56].

Similar to other studies, low temperatures in October, and high age were factors that initiated an early start of hibernation for pregnant females [60], [61]. Bears in colder climates hibernate longer [49]. The temperature in October also correlated negatively with the environmental condition index. In our study area, bears mainly forage on berries in autumn [68]. In late autumn when food availability decreases, the trade-off between energy expenditure and energy consumption might diminish [56]. Older females may have experienced that an early start of hibernation had a positive impact on the energy balance and started to hibernate earlier than younger unexperienced pregnant females. Schooley et al. [69] suggested that pregnant American black bears den after they have stored sufficient fat reserves for winter survival and reproduction in order to avoid being active during periods when food become less abundant.

Pregnant free-ranging bears must cope with more challenging environmental factors than bears in captivity, such as limited food availability, harsh weather conditions, disturbances by humans, or hunting activities. They must gauge the energy costs and benefits of an early denning start. In central Sweden 68% of the presumed pregnant females that had abandoned their dens emerged from their new dens without cubs and 22% of the first dens were abandoned, primarily due to human disturbance [52]. Previous studies have shown that disturbance during hyperphagia and during hibernation period have a negative effect on the bears' fitness and reproductive success [31], [35], [51], [52], [70]. Pregnant females are not protected from hunting, however, they play a crucial role in population growth and start to hibernate earliest [48], [71]–[73]. In our study 47% of the pregnant females started hibernation before the 15 October, the last day hunting is permitted if the quota has not been filled. Therefore, an early start of hibernation could also be a strategy to avoid disturbance and loss of energy during the hunting season. Restricted use of their home range, combined with reduced movements, are known strategies of female brown bears with cubs of the year to avoid male bear encounters during mating season [45], [74]. Several studies have shown that bears try to avoid human disturbance during hibernation, e.g., by selecting den sites far from roads or in concealed and rugged terrain [75]–[80]. Additionally, pregnant females choose better concealed den types, like anthill, soil, and rock dens, than male bears, which often hibernate in open nest dens [81]. Also, previous studies on free-ranging female brown bears in central Sweden have shown that females select predetermined places for denning by visiting their den areas on average more than once a month during season [48]. Male brown bears in the same study area have higher abandonment rates when they had not visited their den sites previously [53]. In our study, during years with good environmental conditions, pregnant females began hibernating earlier rather than using energy reserves for early parturition and lactation, which would have maximized offspring weight at den emergence. During years with bad environmental conditions, the duration of hibernation prior parturition also was shorter. Further research is necessary to determine whether early denning combined with tactically wise denning strategies help pregnant females avoid disturbance. Early start of hibernation has been hypothesized as a strategy for predator avoidance in small mammals [82]. In this regard, it would be important to compare the timing of hibernation and parturition in our hunted population living in a human-dominated landscape with brown bear populations living in areas with low human activities during autumn. In addition, more information about the relationship between female body condition prior to hibernation and the timing of gestation is needed for wild-living bears.
